# Potential Antitumor Mechanism of Propolis Against Skin Squamous Cell Carcinoma A431 Cells Based on Untargeted Metabolomics

**DOI:** 10.3390/ijms252011265

**Published:** 2024-10-19

**Authors:** Jie Wang, Liyuan Cheng, Jingjing Li, Yicong Wang, Siyuan Chen, Zhongdan Wang, Wenchao Yang

**Affiliations:** 1 College of Bee Science and Biomedicine, Fujian Agriculture and Forestry University, Fuzhou 350002, China; wangjie01092023@163.com (J.W.); lijingjing000407@163.com (J.L.); 18265526098@163.com (Y.W.); 18639048028@163.com (S.C.);; 2 College of Food Science, Fujian Agriculture and Forestry University, Fuzhou 350002, China; clyuan0819@163.com

**Keywords:** propolis, metabolomics, skin squamous cell carcinoma, steroid hormone biosynthesis, linoleic acid metabolism

## Abstract

Propolis is a sticky substance produced by honeybees (*Apis mellifera*) through the collection of plant resins, which they mix with secretions from their palate and wax glands. Propolis can inhibit tumor invasion and metastasis, thereby reducing the proliferation of tumor cells and inducing cell apoptosis. Previous research has shown that propolis has an inhibitory effect on skin squamous cell carcinoma A431 cells. Nevertheless, its inhibitory mechanism is unclear because of many significantly different Kyoto Encyclopedia of Genes and Genomes (KEGG) pathways between the ethanol extract of the propolis (EEP) group and the control group of cells. In this study, the main components of EEP and the antitumor mechanism at an IC_50_ of 29.04 μg/mL EEP were determined via untargeted metabolomics determined using ultra high-performance liquid chromatography tandem mass spectrometry (UHPLC‒MS/MS), respectively. The results revealed 43 polyphenolic components in the EEP and 1052 metabolites, with 160 significantly upregulated and 143 significantly downregulated metabolites between cells treated with EEP and solvent. The KEGG enrichment results revealed that EEP significantly inhibited A431 cell proliferation via the steroid hormone biosynthesis and linoleic acid metabolism pathways. These findings may provide valuable insights for the development of targeted therapies for the treatment of cutaneous squamous cell carcinoma.

## 1. Introduction

Propolis is a sticky, solid colloid formed by a mixture of plant resins collected by Western worker bees and secretions from their maxillary glands, wax glands, etc. [1]. There are many known types of propolis worldwide, such as Brazilian green propolis (*Baccharis dracunculifolia* as the main plant source), Brazilian red propolis (*Dalbergia ecastophyllum*), European propolis (*Populus nigra* L.), Russian propolis (*Betula verrucosa Ehrh*), and Cuban and Venezuelan red propolis (*Clusia* spp.) [2]. Their chemical compositions include more than 800 compounds, which are mainly composed of resin (70%), wax (10%), volatile substances (1%), and other organic compounds, including phenolic compounds, esters, flavonoids, terpenes, beta steroids, aromatic aldehydes, alcohols, vitamins, and minerals [3]. Propolis has a wide range of biological activities, such as antibacterial, anti-inflammatory, antioxidant, antitumor, and immune regulatory activities [3].

The antitumor effect of propolis occurs mainly through the regulation of multiple signaling pathways, which include blocking the tumor cell cycle, inducing autophagy and epigenetic regulation, inhibiting tumor invasion and metastasis, inhibiting cancer cell proliferation, and inducing cell apoptosis. Previous studies have shown that propolis has antitumor activity against a range of human cancer cell lines, including oral cancer [4], gastric cancer [5], cervical cancer [6], colon cancer [7], breast cancer [8], and prostate cancer [9] in vitro. Most of these studies have measured the effects of propolis harvested from different geographical regions on the growth, proliferation, and metastasis of cancer cells in vitro [10]. By studying the gene-suppressing potential of 93 propolis components in breast cancer, new potential bioactive compounds in the ethanol extract of propolis (EEP), 3′,4′,7-trihydroxyisoflavone, and baicalein-7-O-β-D glucopyranoside, can bind to *ERα* and *HSP90* to exert anti-breast cancer effects [11]. The cytotoxicity of nanopropolis to human breast cancer cells is greater than that of propolis [12]. In addition, propolis enhances the antitumor effect of 5-fluorouracil in a dose-dependent manner and reduces the side effects on colorectal cancer through intracellular reactive oxygen species production [13]. Ethanol (70%) extract of Cuban red propolis exhibited antiproliferative and cytotoxic effects on MDA MB-231 cells, probably related to PI3K/Akt and ERK1/2 pathways [14]. Ethanol (95%) extract of Chinese propolis has a dose- and time-dependent cytotoxic effect on both MCF-7 (human breast cancer ER(+)) and MDA-MB-231 (human breast cancer ER(-)) cells by inducing apoptosis, regulating the levels of ANXA7, p53, and NF-κB p65, upregulating intracellular ROS, and decreasing mitochondrial membrane potential [15].

Cutaneous squamous cell carcinoma (CSCC), known as squamous cell carcinoma, is a cancer originating from keratinocytes in the epidermis and appendages. This is the leading cause of death among nonmelanoma skin cancers [16]. Squamous cell carcinoma has become a global problem that endangers the physical and mental health of citizens because of its very high incidence and impact on both physical and mental well-being [17]. In recent years, 15 to 35 people have been diagnosed with CSCC per 100,000 people, and its incidence is increasing at a rate of 2% to 4% per year [18]. Studies have shown that itraconazole inhibits the growth of cutaneous squamous cell carcinoma by targeting the 3-hydroxy-3-methylglutaryl-CoA synthase 1 (HMGCS1) and acyl-CoA synthetase long-chain family member 4 (ACSL4) (HMGCS1/ACSL4) axis, depending on the integrated analysis of transcriptomic and proteomic results [19]. Moreover, dihydroartemisinin also inhibits the proliferation, invasion, and migration and promotes apoptosis of A431 cells. Mechanistically, dihydroartemisinin promotes autophagy and modulates the activation of the melanoma II inflammasome pathway and the NF-κB/HIF-1α/VEGF pathway [20]. The proliferation and migration of the CSCC cell line A431 were inhibited by overexpression of *LINC00641* at the cellular level through downregulating the expression of *miR-424*. The same result was also found in the tumor volume in nude mice [21]. The UV-induced apoptosis and doxorubicin-induced apoptosis of CSCC cell lines were more sensitive when E2F7 was inhibited [22]. The survival rate and metastasis of A431 cells are inhibited when *MMP9* in the A431 cell line is inhibited by CRISPR/Cas9-mediated transfection of guide RNA (gRNA), which causes a decrease in viability, migration, and the mRNA expression levels of the oncogenes *TGF-β*, *FGF*, *PI3K*, *VEGF-A,* and vimentin [23]. Our previous research revealed that EEP inhibited the A431 cell line via extracellular matrix (ECM)-receptor interactions, amoebiasis, cell adhesion molecules (CAMs), nonalcoholic fatty liver disease (NAFLD), retrograde endocannabinoid signaling, and Alzheimer’s disease pathways [24]. However, the potential mechanism was also unclear because of complex protein–protein interactions between the differentially expressed proteins. New strategies are needed to explore the potential antitumor mechanism involved.

Metabolomics is an increasingly powerful research tool in the natural sciences and life sciences that has been widely used to elucidate the effects of internal or external stimuli on biological disturbances. Untargeted metabolomics involves the detection of as many metabolites as possible in a single analysis, holding the potential to identify new biomarkers [25]. There are 34 biomarkers related to urine samples from 25 gastric cancer patients and 17 healthy volunteers identified via untargeted metabolomics, which can provide a basis for the early diagnosis of gastric cancer [26]. A total of 31 potential markers of lung tumor cells were also identified via untargeted metabolomics methods, which provides a new method for the discovery and early detection of lung cancer cell markers [27]. In an untargeted metabolomics study of ovarian cancer SKOV3 cells, B-norcholesterol benzimidazole compounds inhibited the intracellular metabolism and protein synthesis and decreased the energy metabolism of SKOV3 cells. These changes lead to the inhibition of proliferation and signal transduction, the elimination of invasiveness and metastasis, and the induction of cell apoptosis, thereby exerting an antitumor effect [28]. The relevance of specific antitumor mechanisms and metabolites of EEP in A431 cells remains to be elucidated.

To address this knowledge gap, ultra high-performance liquid chromatography tandem mass spectrometry (UHPLC‒MS/MS), a cell counting kit-8 (CCK-8), and untargeted metabolomics assays were employed to further reveal the potential antitumor mechanism of propolis against the proliferation of A431 cells.

## 2. Results

### 2.1. Polyphenols of EEP

**Figure 1:**
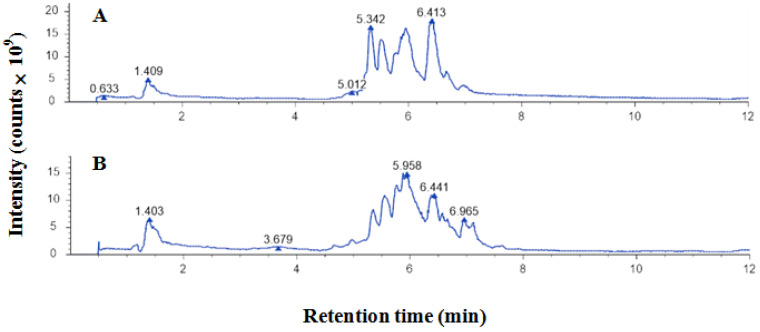
UHPLC‒MS/MS ion spectrum of ethanol-extracted propolis: (**A**) negative ions; (**B**) positive ions.

The UHPLC‒MS/MS ion map of EEP components is shown in Figure 1. There are 43 polyphenolic compounds, the spectra of which are shown in Table 1.

### 2.2. Inhibitory Effect of EEP on the Proliferation of A431 Cells

**Figure 2:**
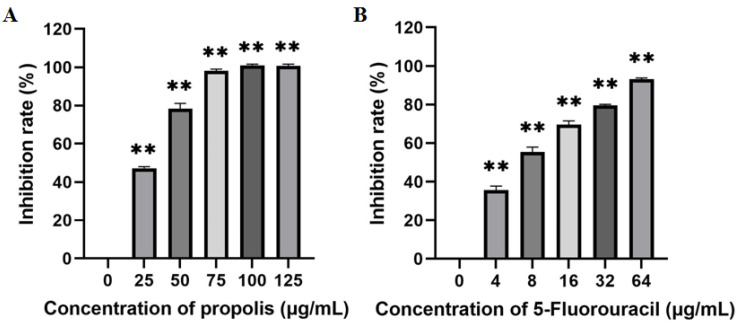
Inhibitory effects of ethanol-extracted propolis (**A**) and 5-fluorouracil (**B**) on the proliferation of A431 cells (there are three biological replicates, each with 6 wells; “**” means significantly different inhibition rates between the treatment group and control group).

EEP inhibited the proliferation of A431 cells in a dose-dependent manner after 48 h of treatment (Figure 2A). The IC_50_ of EEP in A431 cells was 29.04 μg/mL. The IC_50_ of 5-fluorouracil in A431 cells was 7.81 μg/mL (Figure 2B).

### 2.3. Differential Metabolites of A431 Cells Induced by EEP

**Figure 3:**
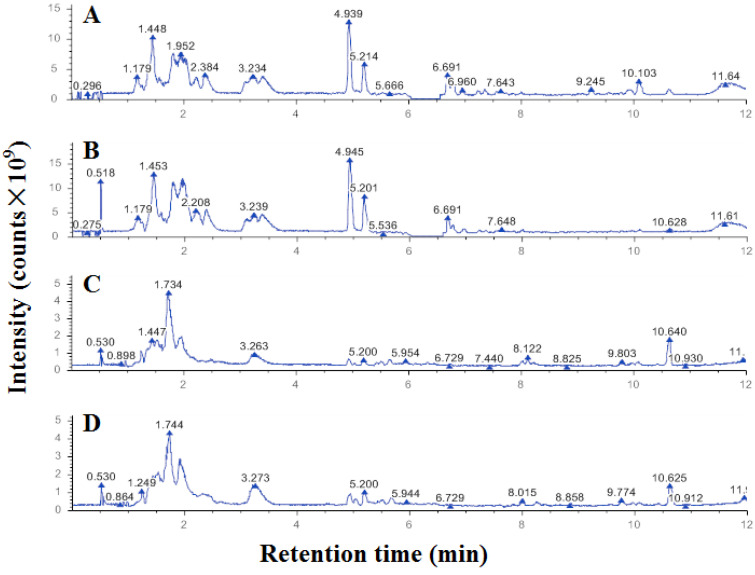
UHPLC‒MS/MS ion spectra of metabolites from A431 cells treated with ethanol-extracted propolis ((**B**) positive and (**D**) negative) and solvent ((**A**) positive and (**C**) negative).

Among the positive ion metabolites (Figure 3A,B), organic acids and their derivatives accounted for 27.15%, and lipids and lipid-like molecules accounted for 26.08%. Among the negative ion metabolites (Figure 3C,D), lipids and lipid-like molecules accounted for 40.51%, and organic acids and their derivatives accounted for 20.25%.

A total of 571 metabolites in positive ion mode and 481 metabolites in negative ion mode were identified in this study. There were 160 metabolites in positive ion mode and 143 metabolites in negative ion mode significantly different between A431 cells in the control group and the propolis group, which were screened according to the criteria of VIP > 1.0, FC > 1.5 or FC < 0.667, and *p* < 0.05 were used (Table 2).

**Figure 4:**
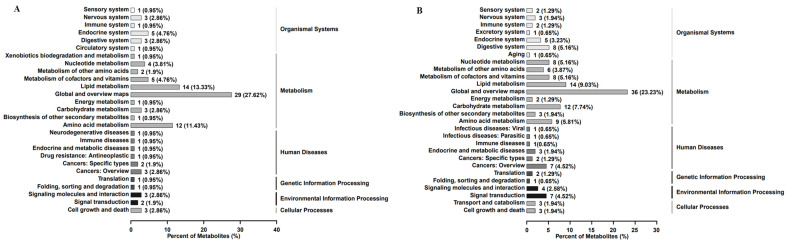
The proportions of Kyoto Encyclopedia of Genes and Genomes (KEGG) pathways enriched with differentially abundant metabolites in A431 cells treated with ethanol-extracted propolis account for the level 1 classification in positive ion mode (**A**) and negative ion mode (**B**).

The proportions of the level 1 classification of differentially abundant metabolites of A431 cells treated with EEP in positive ion mode were as follows: metabolism, 68.57%; organismal systems, 13.33%; human diseases, 8.57%; environmental information processing, 4.76%; cellular processes, 2.86%; and genetic information processing, 1.90% (Figure 4A). The proportions of the level 1 classification of differentially abundant metabolites in negative ion mode were as follows: metabolism, 63.23%; organismal systems, 14.19%; human diseases, 9.68%; environmental information processing, 7.10%; cellular processes, 3.87%; and genetic information processing, 1.94% (Figure 4B).

The significantly different pathways (*p* < 0.05) enriched with differentially abundant metabolites were steroid hormone biosynthesis (both positive and negative ion modes) and linoleic acid metabolism (positive ion mode), the differentially abundant metabolites of which are shown in Table 3.

## 3. Discussion

The polyphenolic components in propolis determined in this study are different from previous reports [24,29]. The difference may be influenced by the extraction procedure, storage, and detection database. Genistein is a flavonoid compound in propolis that can inhibit the growth of prostate cancer cells [30]. Quercetin is a flavonoid in propolis that also induces cell apoptosis [31]. Chrysin, caffeic acid, p-coumaric acid, and ferulic acid are phenolic compounds in propolis that can also induce cell-dependent apoptosis [10,32,33]. These compounds affect the proliferation process of tumor cells.

Propolis has antitumor activity against different cell lines. Brazilian red propolis inhibits the growth of cancer cells. After 24 h of treatment, the IC_50_ values in Hep-2 cells and HeLa cells were 63.48 ± 3.30 µg/mL and 81.40 ± 6.40 µg/mL, respectively [34]. Iranian Ardabil propolis has a dose-dependent toxic effect on both KB and A431 cells. After 48 h incubation, the IC_50_ values of the EEP for the KB and A431 cell lines were 40 ± 8.9 µg/mL and 98 µg/mL, respectively [35]. After 24 h of Polish propolis treatment, the IC_50_ values for tongue cancer cells were approximately 88 µg/mL, 110 µg/mL, and 150 µg/mL [36]. The IC_50_ values of serial samples of Serbian propolis for the human colon cancer cell line HCT-16 were 26.33~143.09 µg/mL [37]. The IC_50_ values of propolis from Thailand for A549 cells were 106 ± 0.004 µg/mL, 199 ± 0.009 µg/mL, and 87 ± 0.012 µg/mL, respectively, and their IC_50_ values for HeLa cells were 81 ± 0.006 µg/mL, 116 ± 0.023 µg/mL, and 54 ± 0.005 µg/mL, respectively [38]. In this study, the IC_50_ of propolis for A431 cells incubated for 48 h was 29.04 μg/mL, which was similar to the previous result of 39.17 μg/mL [24]. This difference may be caused by the representativeness of the raw propolis samples. These different IC_50_ values for different tumor cell lines may be related to the cancer cell type, cancer cell concentration, incubation time, plant source of propolis, propolis extraction process, and storage of propolis.

Propolis has an antitumor effect on A431 cells via extracellular matrix (ECM)-receptor interactions, amoebiasis, cell adhesion molecules (CAMs), nonalcoholic fatty liver disease (NAFLD), retrograde endocannabinoid signaling, and Alzheimer’s disease pathways, as determined by label-free proteomics [24]. However, the interactions among the differentially expressed proteins affected the determination of the potential antitumor mechanism against A431 cells. Untargeted metabolomics was employed to estimate the potential mechanism involved in lung cancer cells and ovarian cancer SKOV3 cells after drug treatment [26,27]. In this study, untargeted metabolomics revealed 1052 small-molecule metabolites, of which 160 metabolites were significantly upregulated and 143 metabolites were significantly downregulated. KEGG enrichment analysis revealed that the two pathways of steroid hormone biosynthesis and linoleic acid metabolism were significantly different.

Among the metabolites enriched in positive ion mode, the significantly enriched pathway was steroid hormone biosynthesis, whose key differentially abundant metabolites were cholesterol, androsterone, estrone, dehydroepiandrosterone (DHEA), and corticosterone. Skin cells contain the entire biochemical apparatus required to produce glucocorticoids, androgens, and estrogens, either from systemic precursors or through the conversion of cholesterol to pregnenolone, which is subsequently converted into biologically active steroids. The differentially abundant metabolite production of this series of steroids by A431 skin cell carcinoma in response to propolis is consistent with this result [39]. Cholesterol is a metabolite associated with multiple biological functions that play complex roles in supporting cancer progression and suppressing immune responses. Preclinical and clinical studies have shown that controlling cholesterol metabolism can inhibit tumor growth, reshape the immune landscape, and enhance antitumor immunity [40]. In our previous study [24], propolis inhibited A431 proliferation by downregulating proteins related to the nonalcoholic fatty liver disease (NAFLD) pathway, which requires activation of the transcription factor sterol regulatory binding protein-1c (SREBP-1c). This NAFLD pathway is induced by dietary cholesterol through activation of the ligand-activated nuclear receptor liver X receptor [41], which is consistent with the downregulation of the metabolite cholesterol in this study. Skin is an androgen target tissue in which 3β-hydroxysteroid dehydrogenase (3β-HSD), 17β-hydroxysteroid dehydrogenase (17β-HSD), and 5α-reductase convert dehydroepiandrosterone, androstenedione, and testosterone into the most effective natural androgen dihydrotestosterone (DHT). This androgen is mainly converted into two phase I metabolites, androstane (ADT) and androstane-3α, 17 β-diol (3α-DIOL) in situ [42]. 3α-DIOL and ADT are then converted into two inactive and easily excreted 3α-DIOL-17 glucuronide (3α-DIOL-17G) and ADT-3 glucuronide (ADT-3G). This metabolic process is also present in prostate cancer [43]. In this study, both dehydroepiandrosterone and androsterone were upregulated, which may be related to this metabolic process after propolis acts on A431 cells.

The biosynthesis of steroid hormones is also a significantly different enrichment pathway in the negative ion mode metabolites, which are tetrahydrocorticosterone, cortodoxone, hydrocortisone, rosterone glucuronide, and adrenosterone. Among them, androsterone is also significantly upregulated, similar to positive ions. Androsterone glucuronide (AoG), a metabolite of circulating androgens under the influence of 5α-reductase activity, is associated with inflammatory lesions [44]. The immune system recognizes and eliminates pathogens and tumor cells, thereby inhibiting tumor growth [45]. Here, the metabolite AoG was downregulated and associated with the inhibition effect on A431 tumor cell proliferation.

The biosynthesis of steroid hormones is also closely related to changes in lipid metabolism and is also a therapeutic intervention point for the treatment of prostate cancer [46,47]. The steroid hormone biosynthesis pathway was the main mechanism of the antitumor effect of *Hericium erinaceus* petroleum ether extract on H22 tumor-bearing mice, according to untargeted metabolomics mechanism analysis [48]. Moreover, apolipoprotein A-1 containing steroid hormones reduces the number of 4,3-keto groups and accelerates the biosynthesis rate of DNA and protein in liver cancer HA-1 mice [49]. Owing to the association between steroidogenesis and breast cancer progression, steroid determination is an important tool for diagnosing breast cancer through the measurement of several steroid hormones and metabolites [50]. Steroid hormones can also regulate the expression of transforming growth factor and epidermal growth factor receptors in endometrial cancer cells to inhibit their proliferation [51].

Another significantly different pathway in positive ion mode is the linoleic acid metabolism pathway, which is enriched with differentially abundant metabolites, such as phosphatidylcholine (PC32:1), 12, 13-dihydroxy-9Z-octadecenoic acid ((+/−)12(13)-DiHOME), and 13-hydroperoxylinoleic acid (13-HpODE). It was reported that the increased choline consumption and phosphatidylcholine secretion of cancer cells were positively correlated with the cell proliferation rate. Phosphatidylcholine levels increased during the malignant transformation of human breast and prostate epithelial cells [52]. Lipid peroxidation processes involve linoleic acid in the production of 13-HpODE [53], while the source of choline is the degradation of choline-containing lipids [51]. Linoleic acid, which has important functions in health and disease, helps stimulate skin growth, maintain bone health, regulate metabolism, maintain reproductive system function, and enhance the metabolic adaptability and antitumor immunity of CD8^+^ T cells [54]. Linoleic acid can significantly inhibit the proliferation of hepatocellular carcinoma through metabolomics assessment and can also exhibit anti-proliferative and anti-invasive activities in endometrial cancer cell lines [55,56]. Metabolomic analysis of patients with precursor B-cell acute lymphoblastic leukemia revealed that the linoleic acid metabolic disorder pathway is closely related to B-cell acute lymphoblastic leukemia [57]. The linoleic acid metabolic pathway was also one of the five metabolic pathways affected in the orthotopic lung tumor model [58]. The dihydroxyflavone pinothiocyanate from plants and propolis inhibited human ileocecal colorectal adenocarcinoma OC43 cells via the linoleic acid and arachidonic acid metabolic axis through the AHR/CYP1A1 pathway [59]. In this study, untargeted metabolomics revealed the ability of propolis to inhibit the proliferation of A431 cells via the steroid hormone biosynthesis and linoleic acid metabolic pathways.

Some differentially abundant metabolites are components of EEP, which has a direct inhibitory effect on cancer cells. Chrysin, one of the significantly upregulated differentially abundant metabolites, has anticancer effects by regulating the apoptosis of breast cancer, gastrointestinal cancer, liver cancer, hepatocellular carcinoma, and bladder cancer [33,60]. Glycine sojae is an isoflavone and another upregulated metabolite in A431 cells treated with propolis. It also has significant cytotoxic effects on human gastric cancer cells by regulating cycle-related proteins [61]. Upregulated metabolite naringenin is a bioactive polyphenol that can inhibit the development of cancer in various parts of the body. Its anticancer activity is pleiotropic, including regulating different cell signaling pathways, inhibiting the production of cytokines and growth factors, and arresting the cell cycle [62]. Chrysin, glycine sojae, and naringenin in propolis are absorbed by cancer cells, thereby causing the apoptosis of A431 cells.

There are several other metabolites associated with anticancer activity. Sphingomyelin metabolism produces anticancer signals such as ceramide and sphingosine, which may inhibit cell proliferation and induce differentiation and apoptosis in colon cancer [63]. The metabolite sphingomyelin was also significantly upregulated in this study. The formation and development of adenoma is caused by excessive autonomous secretion of aldosterone [64]. The main metabolite of aldosterone is tetrahydroaldosterone [65], whose downregulation can reflect a decrease in aldosterone content. This downregulation of tetrahydroaldosterone was also observed in this study. L-palmitoylcarnitine was also downregulated in this study, which is consistent with the detection of metabolites of breast cancer cells inhibited by berberine in the hypoxic microenvironment [66].

## 4. Materials and Methods

### 4.1. Propolis Extraction and Polyphenols Determination

The raw poplar propolis sample was the same as that used in our previous report [29]. The extraction procedure for EEP was modified [29]. Briefly, 133.3 g of crushed propolis powder was dissolved in 1000 mL of a 70% ethanol solution. The mixture was stirred for 2 h and then placed in an ultrasonic cleaner (40 kHz, 60 min, 30 °C water bath). The mixture was filtered under vacuum after incubation at room temperature for 48 h, and then it was centrifuged at 8000× *g* for 10 min at 4 °C. These above operations were repeated three times. The ethanol in the supernatant was evaporated in a fume hood for 48 h and dried in a constant-temperature drying oven at 60 °C. The propolis extract was collected and stored in a −30 °C refrigerator for further experiments.

The chemical components of EEP were determined via an untargeted metabolomics method by a UHPLC–MS/MS system [Vanquish UHPLC system (Thermo Fisher Scientific Inc., Germering, Germany) coupled with a Q Exactive^TM^ HF/Q Exactive^TM^ HF-X mass spectrometer (ThermoFisher, Germering, Germany)], which is performed by Novogene Co., Ltd. (Beijing, China). In brief, 0.1 g of EEP was dissolved in 500 μL of 80% methanol aqueous solution in an EP tube. The mixture was vortexed and then placed in an ice bath for 5 min. The mixture was subsequently centrifuged at 15,000× *g* at 4 °C for 20 min (D3024R, Scilogex, Rocky Hill, CT, USA). The supernatant was diluted with a 53% methanol aqueous solution and then centrifuged again at 15,000× *g* and 4 °C for 20 min. The supernatant was injected into the UHPLC–MS/MS system for component determination. A Hypersil Gold column (C18, 100 × 2.1 mm, 1.9 μm, Thermo Scientific, Waltham, MA, USA) with mobile phases A (0.1% formic acid) and B (methanol) was employed. The gradient gradually changed from 98% A to 15%, 0, and 98% at the 3rd, 10th, and 10.1th minutes to 12 min at 98% A with a flow rate of 200 μL/min. The Q Exactive^TM^ HF/Q Exactive^TM^ HF-X mass spectrometer was operated in positive/negative polarity mode with a spray voltage of 3.5 kV, capillary temperature of 320 °C, sheath gas flow rate of 35 psi, aux gas flow rate of 10 L/min, S-lens RF level of 60, and aux gas heater temperature of 350 °C. MS/MS secondary scans are data-dependent scans.

The raw data files obtained from UHPLC–MS/MS were processed via Compound Discoverer 3.3 (CD3.3, Thermo Fisher) software to perform peak alignment, peak picking, and quantitation for each metabolite, whose main parameters were set as follows: retention time tolerance of 0.2 min; actual mass tolerance of 5 ppm; signal intensity tolerance of 30%; signal/noise ratio of 3; and minimum intensity. The peak area was quantified, and the target ions were integrated. The molecular formula was predicted through the molecular ion peak and fragment ions and comparison with mzCloud. The background ions were removed from the blank samples. The original quantitative results were standardized according to the following formula: sample original quantitative value/(sum of sample metabolite quantitative values/sum of quality control (QC)1 sample metabolite quantitative values) to obtain the relative peak area. The compounds with a coefficient of variation (CV) of relative peak area greater than 30% in the QC sample were deleted. The identification and relative quantitative results of the metabolites were obtained. Polyphenols were selected according to the secondary classification of the components.

### 4.2. Antitumor Effects of Propolis on the Proliferation of A431 Cells

The human skin squamous cell carcinoma A431 cell line (purchased from Wuhan Purosai Life Sciences Co., Ltd., Wuhan, China) was cultured with a special complete culture or serum-free cell freezing medium (Wuhan Purosai Life Sciences Co., Ltd.) in a 5% CO_2_ humidified incubator at 37 °C (C150, Binder, Tuttlingen, Germany).

After the cells were washed 2–3 times with phosphate-buffered saline (PBS, pH 7.2–7.4, Sinopharm Chemical Reagent Co., Ltd., Shanghai, China), they were digested with 1 mL of 0.25% trypsin (HyClone, Thermo Scientific, Waltham, MA, USA). The digestion was terminated with the complete culture medium. The digested cells were subsequently centrifuged at 137× *g* for 5 min and resuspended using the complete culture medium in a centrifuge tube. The cells were diluted with the complete culture medium to a concentration of 5 × 10^4^ cells/mL by staining count with 0.4% trypan blue (Beijing Solarbio Science & Technology Co., Ltd., Beijing, China). A cell suspension (0.1 mL) was added to each well of a 96-well plate, which was placed in a cell culture incubator at 37 °C and 5% CO_2_ for 24 h.

EEP (0.1 g) was dissolved in a mixture of 0.5 mL of dimethyl sulfoxide (DMSO, Sinopharm Chemical Reagent Co., Ltd., Shanghai, China) and 9.5 mL of complete cell culture medium. This solution was subpackaged and placed in a −20 °C refrigerator for further use. During the experiment, the propolis solution was diluted with the complete culture medium to the required concentration for further experiments.

The EEP solutions were diluted to 25, 50, 75, 100, and 125 μg/mL in a complete culture medium. The concentrations of 5-fluorouracil (HPLC grade; purchased from Sigma-Aldrich Co., St. Louis, MO, USA) solution used were 4, 8, 16, 32, and 64 μg/mL complete culture medium. The complete culture medium containing 0.0625% DMSO (equal to the quantity of DMSO in 125 μg/mL EEP solution) was used as the control group. There were triplicate and six duplicate wells for each treatment. The treatments and control group solutions (100 μL) were added and then cultured in the cell culture incubator for 48 h. The cells were washed with PBS, and then 110 μL of complete culture medium containing 10 μL of CCK-8 solution (DOJINDO, Kumamoto, Japan) was added in the dark. The absorbances at 450 nm were determined via a microplate reader (1510, Thermo Fisher Waltham, MA, USA) after 2 h of incubation in a cell culture incubator. The proliferation inhibition rate (%) was calculated as [(OD of control wells having cells and culture medium containing CCK-8 − OD of experimental wells having different concentrations of propolis solutions)/(OD of control wells having cells and culture medium containing CCK-8 − OD of blank wells containing CCK-8 and cells and culture medium free)] × 100%.

### 4.3. Untargeted Metabolomic Effects of Propolis on the Proliferation of A431 Cells

The cells were added to a 6-well plate after cell counting. They were treated with the complete culture medium containing 0.0625% DMSO (equal to DMSO in 125 μg/mL EEP solution) and the IC_50_ concentration of EEP (29.04 μg/mL) after 24 h of incubation. These cells were washed with 4 °C PBS (pH 7.2–7.4) 3 times after 48 h of incubation. After digestion with 0.25% trypsin, the cells were washed with 4 °C PBS (pH 7.2–7.4) 3 times again. These cells were transferred in a 1.5 mL centrifuge tube and quickly frozen with liquid nitrogen for 15 min. They were stored at −80 °C.

Aqueous methanol (80%, 300 μL) was added to the cells. Then, the samples were put into liquid nitrogen for quick freezing for 5 min. The frozen cells were thawed on ice and vortexed for 30 s. The samples were treated with the assistance of an ultrasonic cleaner for 6 min and centrifuged at 2560× *g* at 4 °C for 1 min. The supernatant was transferred into a new centrifuge tube and freeze-dried. A 10% methanol solution was used to dissolve the powder. The solution was injected into the UHPLC‒MS/MS system for analysis.

Each experiment had six replicates, and equal volumes of samples were taken from each experimental sample and mixed as QC samples. A 53% methanol aqueous mixture was used as a blank sample. The pretreatment procedure for the blank sample was the same as that for the experimental samples.

The conditions of the UHPLC‒MS/MS system are the same as those used for the determination of propolis components described above.

### 4.4. Statistical Analysis

All the experiments were performed in triplicate except for untargeted metabolomic effects of propolis on the proliferation of A431 cells, and the results are expressed as the means ± standard errors. The cell death (%) was transformed to arcsin (degree) values (according to the formula: arc sin√p) before ANOVA, which was performed via GraphPad Prism 9.5.1 for Windows (GraphPad Software, Inc. San Diego, CA, USA) to analyze the significance of differences (*p* < 0.01: extremely statistically significant differences between the different treatment groups; *p* < 0.05: statistically significant differences).

The identified metabolites were annotated via the KEGG database. For multivariate statistical analysis, the metabolomics data processing software metaX was used to transform the data, and then principal component analysis (PCA) and partial least squares discriminant analysis (PLS-DA) were performed to obtain the VIP value of each metabolite. In the univariate analysis, the statistical significance (*p*-value) of each metabolite between the two groups was calculated via a *t*-test, and the fold change (FC value) of the metabolite between the two groups was calculated. The default criteria for differentially abundant metabolite screening are VIP > 1, *p* < 0.05, and FC ≥ 1.5 or FC ≤ 0.667.

## 5. Conclusions

There was a total of 43 polyphenolic components in the ethanol extraction of propolis (EEP), of which the IC_50_ in A431 cells was 29.04 μg/mL. Untargeted metabolomics revealed 1052 small-molecule metabolites, of which 160 were significantly upregulated and 143 were significantly downregulated. The KEGG enrichment results revealed that EEP significantly inhibited A431 cell proliferation via the steroid hormone biosynthesis and linoleic acid metabolism pathways. These results provide evidence for the development of targeted drugs for the treatment of cutaneous squamous cell carcinoma.

## Figures and Tables

**Table 1:** Polyphenols (flavonoids ID 1–24 and phenols ID 25–43) of ethanol-extracted propolis determined by untargeted metabolomics.

ID	Name	Formula	Molecular Weight	RT/[min]	*m*/*z*	Relative Quantitative Value	Polarity Ion Mode
1	7-Methoxyflavone	C_16_H_12_O_3_	252.0743	2.319	253.0816	165,691,937.9	positive
2	4′-Methoxyflavone	C_16_H_12_O_3_	252.0742	2.751	253.08127	40,927,754.05	positive
3	Eriodictyol	C_15_H_12_O_6_	242.0573	5.114	287.05548	19,766,216.42	negative
4	Taxifolin	C_15_H_12_O_7_	304.0579	5.17	305.06513	33,877,502.65	positive
5	Neodiosmin	C_28_H_32_O_15_	608.1722	5.334	609.17948	25,006,731.57	positive
6	Hesperetin	C_16_H_14_O_6_	302.0782	5.406	320.11203	98,982,625.12	positive
7	Rutin	C_27_H_30_O_16_	610.1532	5.463	609.14589	60,165,204.17	negative
8	Quercetin-3β-D-glucoside	C_21_H_20_O_12_	464.0958	5.497	463.0885	150,118,518.8	negative
9	Epicatechin	C_15_H_14_O_6_	290.0793	5.547	289.07202	205,621,918.1	negative
10	Nobiletin	C_21_H_22_O_8_	442.1237	5.555	425.12034	38,494,781.4	positive
11	Trifolin	C_21_H_20_O_11_	448.0999	5.59	449.10716	129,227,583.4	positive
12	Galangin	C_15_H_10_O_5_	270.0526	5.784	271.05985	6,988,985,974	positive
13	Quercetin	C_15_H_10_O_7_	302.0424	5.808	301.03512	12,911,294,036	negative
14	Kaempferol	C_15_H_10_O_6_	286.0477	6.025	285.04042	3,447,864,851	negative
15	Apigenin	C_15_H_10_O_5_	270.0526	6.048	271.05989	7,294,339,651	positive
16	Sakuranin	C_22_H_24_O_10_	470.1182	6.36	471.12546	148,046,024.5	positive
17	Pinocembrin	C_15_H_12_O_4_	256.0733	6.452	257.0806	3,402,672,117	positive
18	Chrysin	C_15_H_10_O_4_	254.0577	6.606	255.06497	9,396,111,669	positive
19	Kaempferitrin	C_27_H_30_O_14_	578.159	6.657	579.16626	15,900,785.8	positive
20	Wogonin	C_16_H_12_O_5_	284.0682	6.773	285.07551	1,690,423,123	positive
21	Luteolin	C_15_H_10_O_6_	286.0477	6.95	285.04047	1,048,387,356	negative
22	Naringenin	C_15_H_12_O_5_	272.0685	6.959	271.06118	6,956,970,847	negative
23	Vitexin	C_21_H_20_O_10_	454.0871	6.968	455.09441	328,176,072.8	positive
24	Myricitrin	C_21_H_20_O_12_	464.0945	9.333	463.08725	1,004,733.206	negative
25	Metanephrine	C_10_H_15_NO_3_	197.105	1.782	198.11208	148,715,444.3	positive
26	Epinephrinebitartrate	C_13_H_19_NO_9_	333.105	1.99	334.11243	51,641,235.49	positive
27	Vanillin	C_8_H_8_O_3_	130.063	4.603	131.07061	12,412,803.31	positive
28	2-Methoxyresorcinol	C_7_H_8_O_3_	140.047	4.908	141.05468	49,647,059.53	positive
29	Catechol	C_6_H_6_O_2_	110.037	5.035	109.02963	221,250,004.2	negative
30	Vanillylalcohol	C_8_H_10_O_3_	154.063	5.129	153.05594	167,498,687.3	negative
31	N-Acetyldopamine	C_10_H_13_NO_3_	195.09	5.143	196.09692	253,902,426.9	positive
32	3-Methoxytyramine	C_9_H_13_NO_2_	167.095	5.225	202.06389	3,554,901.715	negative
33	L-Adrenaline	C_9_H_13_NO_3_	183.089	5.23	184.09673	39,891,603.29	positive
34	Hydroquinone	C_6_H_6_O_2_	110.037	5.26	111.04412	142,215,214.3	positive
35	Homovanillicacid	C_9_H_10_O_4_	182.058	5.266	181.05065	99,031,088.98	negative
36	4-Methylphenol	C_7_H_8_O	108.058	5.33	107.05028	64,119,942.28	negative
37	Isoproterenol	C_11_H_17_NO_3_	211.12	5.55	210.11346	23,052,347.25	negative
38	Eugenol	C_10_H_12_O_2_	164.084	5.598	163.07669	141,167,860.1	negative
39	o-Cresol	C_7_H_8_O	108.057	5.617	109.06481	32,449,852.97	positive
40	4-Nitrophenol	C_6_H_5_NO_3_	139.027	5.796	138.0198	83,038,733.11	negative
41	4-Butylresorcinol	C_10_H_14_O_2_	166.099	5.829	149.09605	103,300,893.4	positive
42	Phloroglucinol	C_6_H_6_O_3_	126.032	6.064	125.02453	1,391,500,576	negative
43	Pyrogallol	C_6_H_6_O_3_	126.032	11.989	127.039	95,430,031.51	positive

**Table 2:** Significantly differentially abundant metabolites between A431 cells in the control group and the ethanol-extracted propolis group (*p* < 0.05).

ID	Name	Formula	Molecular Weight	RT/[min]	*m*/*z*	*p* Value	Up/Downregulated	Polarity Mode
1	(3R)-8-hydroxy-3-(4-methoxyphenyl)-3,4-dihydro-1H-2-benzopyran-1-one	C_16_H_14_O_4_	270.08975	6.435	269.08247	1.02 × 10^−14^	up	negative
2	(+/−)12(13)-DiHOME	C_18_H_34_O_4_	296.2349	8.268	319.22416	1.08 × 10^−11^	up	positive
3	Maslinicacid	C_30_H_48_O_4_	494.33677	8.303	495.34404	7.59 × 10^−10^	up	positive
4	(2R)-5-hydroxy-7-methoxy-2-phenyl-3,4-dihydro-2H-1-benzopyran-4-one	C_16_H_14_O_4_	270.08863	7.197	271.0959	8.28 × 10^−10^	up	positive
5	Genistein	C_15_H_10_O_5_	270.05336	6.733	269.04608	1.6 × 10^−9^	up	negative
6	5,6-dimethoxy-2-(2-methoxyphenyl)-4H-chromen-4-one	C_18_H_16_O_5_	294.08863	6.858	295.09591	1.86 × 10^−9^	up	positive
7	Naringenin	C_15_H_12_O_5_	272.06895	5.481	271.06168	2.52 × 10^−9^	up	negative
8	trans-10-Heptadecenoicacid	C_17_H_32_O_2_	314.24627	7.167	313.23899	2.67 × 10^−9^	up	negative
9	4-methoxy-6-[2-(4-methoxyphenyl)ethyl]-2H-pyran-2-one	C_15_H_16_O_4_	282.08876	8.337	283.09604	3.85 × 10^−9^	up	positive
10	Pinocembrin	C_15_H_12_O_4_	256.07376	5.918	257.08104	4.47 × 10^−9^	up	positive
11	Adrenosterone	C_19_H_24_O_3_	300.17302	8.198	299.16574	7.19 × 10^−9^	up	negative
12	12-epiLeukotrieneB4	C_20_H_32_O_4_	318.21778	8.275	363.216	7.82 × 10^−9^	up	negative
13	Tetrahydroaldosterone	C_21_H_32_O_5_	364.22327	8.263	363.216	9.32 × 10^−9^	up	negative
14	N-[2-acetyl-5-(tert-butyl)-3-thienyl]-2-(propylsulfanyl)nicotinamide	C_19_H_24_N_2_O_2_S_2_	376.13179	6.844	375.12451	2.25 × 10^−8^	up	negative
15	SM8:1;2O/12:0	C_25_H_51_N_2_O_6_P	506.34854	8.729	529.33785	2.6 × 10^−8^	up	positive
16	Isorhapontigenin	C_15_H_14_O_4_	258.08978	6.925	257.08251	2.73 × 10^−8^	up	negative
17	4-chloro-5-morpholino-2-quinoxalin-2-ylpyridazin-3(2H)-one	C_16_H_14_ClN_5_O_2_	343.084	2.011	344.09127	4.17 × 10^−8^	up	positive
18	Papaverine	C_20_H_21_NO_4_	339.14752	5.87	340.15479	4.44 × 10^−8^	up	positive
19	N-Acetylsphingosine	C_20_H_39_NO_3_	363.27507	9.628	364.28235	7.49 × 10^−8^	up	positive
20	L-cysteine	C_3_H_7_NO_2_S	121.01995	1.509	122.02727	9.54 × 10^−8^	up	positive
21	Chrysin	C_15_H_10_O_4_	254.05827	6.656	253.051	1.02 × 10^−7^	up	negative
22	Dehydroepiandrosterone(DHEA)	C_19_H_28_O_2_	270.1984	9.61	271.20554	1.13 × 10^−7^	up	positive
23	methyl3,4,5-trihydroxycyclohex-1-ene-1-carboxylate	C_8_H_12_O_5_	210.05314	8.341	211.06041	1.48 × 10^−7^	up	positive
24	gamma-Glutamylcysteine	C_8_H_14_N_2_O_5_S	250.06268	1.657	249.05541	1.71 × 10^−7^	up	negative
25	1-Methyladenosine	C_11_H_15_N_5_O_4_	281.11189	1.883	282.11917	1.72 × 10^−7^	up	positive
26	Hydrocortisone	C_21_H_30_O_5_	362.20763	7.825	361.20035	2.4 × 10^−7^	up	negative
27	Naringeninchalcone	C_15_H_12_O_5_	272.06889	5.493	273.07612	2.69 × 10^−7^	up	positive
28	2-oxo-2H-chromene-3-carboxylicacid	C_10_H_6_O_4_	190.0264	6.86	191.03368	3.02 × 10^−7^	up	positive
29	H-Gly-Pro-OH	C_7_H_12_N_2_O_3_	172.08505	1.983	171.0777	3.39 × 10^−7^	up	negative
30	Resveratrol	C_14_H_12_O_3_	228.07915	6.837	227.07188	3.39 × 10^−7^	up	negative
31	N-Methylhydantoin	C_4_H_6_N_2_O_2_	114.04328	1.739	115.05055	3.59 × 10^−7^	down	positive
32	4-MethoxycinnamicAcid	C_10_H_10_O_3_	178.06217	7.416	379.11392	4.6 × 10^−7^	up	positive
33	D-Glucosamine6-phosphate	C_6_H_14_NO_8_P	259.04597	1.454	260.05325	6.57 × 10^−7^	up	positive
34	ProstaglandinA1ethylester	C_22_H_36_O_4_	346.25129	9.137	345.24402	8.48 × 10^−7^	up	negative
35	5,7-dihydroxy-3-(4-hydroxyphenyl)-4H-chromen-4-one	C_15_H_10_O_5_	270.05304	5.746	271.06031	9.09 × 10^−7^	up	positive
36	4-[2-(2-oxo-1-imidazolidinyl)ethyl]-1lambda~6~,4-thiazinane-1,1-dione	C_9_H_17_N_3_O_3_S	248.10518	6.943	247.0979	9.44 × 10^−7^	up	negative
37	(+/−)-Equol	C_15_H_14_O_3_	242.09476	7.083	241.08748	1.02 × 10^−6^	up	negative
38	Piceatannol	C_14_H_12_O_4_	244.07412	6.713	243.06685	1.06 × 10^−6^	up	negative
39	Ursolicacid	C_30_H_48_O_3_	456.36128	9.427	455.354	1.28 × 10^−6^	up	negative
40	Glycerol1-hexadecanoate	C_19_H_38_O_4_	330.27757	8.603	329.27033	1.31 × 10^−6^	up	negative
41	Glycitein	C_16_H_12_O_5_	284.06896	6.828	283.06168	1.32 × 10^−6^	up	negative
42	Mag(18:1)	C_21_H_40_O_4_	356.29292	10.356	357.30024	1.35 × 10^−6^	down	positive
43	5-hydroxy-6,7-dimethoxy-2-phenyl-4H-chromen-4-one	C_17_H_14_O_5_	280.0731	7.82	281.08037	1.93 × 10^−6^	up	positive
44	Luteolin	C_15_H_10_O_6_	286.04863	6.035	285.04137	2.55 × 10^−6^	up	negative
45	SM8:0;2O/12:0	C_25_H_53_N_2_O_6_P	508.36466	9.391	531.35384	2.69 × 10^−6^	up	positive
46	Cys-Gly	C_5_H_10_N_2_O_3_S	178.04139	1.703	177.03412	2.69 × 10^−6^	up	negative
47	ChlorogenicAcidMethylEster	C_17_H_20_O_9_	368.11135	5.712	367.10407	2.95 × 10^−6^	up	negative
48	Ethyloleate	C_20_H_38_O_2_	310.2871	9.807	311.2943	3.03 × 10^−6^	up	positive
49	Thiazolidine-4-carboxylicacid	C_4_H_7_NO_2_S	133.01974	1.501	134.02701	3.04 × 10^−6^	up	positive
50	P-AminohippuricAcid	C_9_H_10_N_2_O_3_	194.06932	3.142	195.07649	3.46 × 10^−6^	down	positive
51	Acetyl-L-carnitine	C_9_H_17_NO_4_	203.11608	1.768	204.12335	3.6 × 10^−6^	down	positive
52	JWH250N-pentanoicacidmetabolite	C_22_H_23_NO_4_	401.13942	5.25	400.13214	3.72 × 10^−6^	up	negative
53	Hexanoylcarnitine	C_13_H_25_NO_4_	259.17874	5.529	260.18602	3.97 × 10^−6^	down	positive
54	2-Butoxyaceticacid	C_6_H_12_O_3_	132.07877	5.546	131.07149	5.21 × 10^−6^	down	negative
55	LPGO-20:1	C_26_H_53_O_8_P	524.34837	9.656	523.34109	5.42 × 10^−6^	up	negative
56	(+/−)9-HpODE	C_18_H_32_O_4_	312.23068	7.109	311.2234	6.15 × 10^−6^	up	negative
57	Betulin	C_30_H_50_O_2_	442.38096	10.323	443.38802	7.56 × 10^−6^	up	positive
58	gamma-Glutamylleucine	C_11_H_20_N_2_O_5_	260.13759	5.363	259.13031	8.38 × 10^−6^	up	negative
59	Phenylglyoxylicacid	C_8_H_6_O_3_	150.03189	5.84	301.07104	9.12 × 10^−6^	up	positive
60	Formononetin	C_16_H_12_O_4_	268.07286	7.422	269.08017	9.19 × 10^−6^	up	positive
61	Wogonin	C_16_H_12_O_5_	284.06782	6.827	285.07514	9.35 × 10^−6^	up	positive
62	Meclocycline	C_22_H_21_ClN_2_O_8_	476.09669	6.087	475.08941	9.69 × 10^−6^	up	negative
63	Hematoxylin	C_16_H_14_O_6_	302.07953	5.493	301.07225	1.03 × 10^−5^	up	negative
64	5,7-dihydroxy-6-methoxy-2-phenyl-3,4-dihydro-2H-1-benzopyran-4-one	C_16_H_14_O_5_	124.06416	5.93	287.09149	1.06 × 10^−5^	up	positive
65	4-Hydroxy-3-methoxyphenylglycolsulfate	C_9_H_12_O_7_S	264.03101	4.801	263.02373	1.06 × 10^−5^	up	negative
66	RKK	C_18_H_38_N_8_O_4_	408.32427	10.656	431.31357	1.13 × 10^−5^	down	positive
67	Υ-Glutamylcysteine	C_8_H_14_N_2_O_5_S	250.06246	1.994	251.06973	1.19 × 10^−5^	up	positive
68	gamma-Glutamyltyrosine	C_14_H_18_N_2_O_6_	310.1163	4.941	311.12358	1.32 × 10^−5^	up	positive
69	SPB15:0;2O	C_15_H_33_NO_2_	259.25039	6.784	260.25765	1.58 × 10^−5^	down	positive
70	(6E,10E)-3,7,11,15-tetramethylhexadeca-1,6,10,14-tetraene-3,5,9-triol	C_20_H_34_O_3_	339.27689	8.98	362.26611	1.75 × 10^−5^	up	positive
71	Benzyl6-O-beta-D-glucopyranosyl-beta-D-glucopyranoside	C_19_H_28_O_11_	478.16356	7.12	477.15628	1.8 × 10^−5^	up	negative
72	1-(4-hydroxyphenyl)propane-1,2-diol	C_9_H_12_O_3_	208.07353	8.381	191.07051	1.9 × 10^−5^	up	positive
73	23-Nordeoxycholicacid	C_23_H_38_O_4_	378.27712	9.48	401.26638	2.04 × 10^−5^	down	positive
74	18-β-Glycyrrhetinicacid	C_30_H_46_O_4_	470.3403	9.021	469.33302	2.1 × 10^−5^	up	negative
75	Acetylcysteine	C_5_H_9_NO_3_S	163.03053	1.535	146.02726	2.17 × 10^−5^	up	positive
76	KKK	C_18_H_38_N_6_O_4_	384.28518	10.175	367.28192	2.2 × 10^−5^	down	positive
77	Kynurenicacid	C_10_H_7_NO_3_	189.0429	2.602	190.05017	2.26 × 10^−5^	down	positive
78	Dibutylsebacate	C_18_H_34_O_4_	314.24488	7.159	337.23407	2.46 × 10^−5^	up	positive
79	4-Oxoproline	C_5_H_7_NO_3_	129.04281	2.212	128.03553	2.66 × 10^−5^	up	negative
80	Nonadecanoicacid	C_19_H_38_O_2_	298.28674	8.904	321.27596	2.69 × 10^−5^	up	positive
81	(2R,3S,4S,5R,6R)-2-(hydroxymethyl)-6-(propan-2-yloxy)oxane-3,4,5-triol	C_9_H_18_O_6_	244.0882	5.68	245.09547	2.78 × 10^−5^	up	positive
82	Tetranor-12(S)-HETE	C_16_H_26_O_3_	288.16699	7.738	289.17427	2.85 × 10^−5^	down	positive
83	cyclohexyl{4-[4-nitro-2-(1H-pyrrol-1-yl)phenyl]piperazino}methanone	C_21_H_26_N_4_O_3_	404.18011	6.99	405.18737	2.98 × 10^−5^	down	positive
84	N-Acetyl-L-cysteine	C_5_H_9_NO_3_S	163.0306	3.649	164.03786	3.22 × 10^−5^	up	positive
85	1-Palmitoylglycerol	C_19_H_38_O_4_	330.27714	9.892	353.26637	3.46 × 10^−5^	down	positive
86	Normorphine	C_16_H_17_NO_3_	271.12447	5.687	272.13175	3.69 × 10^−5^	up	positive
87	DL-Lysine	C_6_H_14_N_2_O_2_	146.10571	1.213	147.11295	3.76 × 10^−5^	down	positive
88	23-Norcholicacid	C_23_H_38_O_5_	394.27029	10.275	393.26302	3.88 × 10^−5^	up	negative
89	2,3-dihydroxypropyl12-methyltridecanoate	C_17_H_34_O_4_	284.235	9.022	285.24228	4.19 × 10^−5^	down	positive
90	Ethyl-β-D-glucuronide	C_8_H_14_O_7_	222.07419	2.183	221.06691	4.2 × 10^−5^	down	negative
91	N-Acetylasparticacid	C_6_H_9_NO_5_	175.04819	1.64	174.04091	4.39 × 10^−5^	up	negative
92	6-(3-hydroxybutan-2-yl)-5-(hydroxymethyl)-4-methoxy-2H-pyran-2-one	C_11_H_16_O_5_	266.05726	6.879	267.06454	4.66 × 10^−5^	up	positive
93	DL-4-Hydroxyphenyllacticacid	C_9_H_10_O_4_	200.06866	5.193	181.05081	4.73 × 10^−5^	up	negative
94	Metanephrine	C_10_H_15_NO_3_	197.10607	5.366	198.11334	5.41 × 10^−5^	up	positive
95	TPH	C_15_H_23_N_5_O_5_	335.15945	5.853	336.16667	5.66 × 10^−5^	up	positive
96	4-(2-((4-Cyanophenyl)amino)oxazol-5-yl)benzonitrile	C_17_H_10_N_4_O	286.08461	5.45	285.07733	5.73 × 10^−5^	up	negative
97	3-Hydroxydecanoicacid	C_10_H_20_O_3_	188.14149	6.734	187.13421	5.85 × 10^−5^	down	negative
98	Indole-3-lacticacid	C_11_H_11_NO_3_	205.07406	5.613	204.06678	6.04 × 10^−5^	up	negative
99	Phenylpyruvicacid	C_9_H_8_O_3_	164.04762	5.193	163.04035	6.06 × 10^−5^	up	negative
100	1-Methylguanine	C_6_H_7_N_5_O	165.06557	4.74	166.07284	6.59 × 10^−5^	up	positive
101	(±)5(6)-DiHET	C_20_H_34_O_4_	320.23286	9.761	321.24006	7.25 × 10^−5^	down	positive
102	Monoolein	C_21_H_40_O_4_	356.29294	10.106	379.28212	7.32 × 10^−5^	down	positive
103	SM12:2;2O/8:0	C_25_H_49_N_2_O_6_P	550.33985	8.586	549.33257	8.46 × 10^−5^	up	negative
104	L-Palmitoylcarnitine	C_23_H_45_NO_4_	399.33528	10.351	400.34256	9.52 × 10^−5^	down	positive
105	Ureidosuccinicacid	C_5_H_8_N_2_O_5_	176.04359	1.49	175.03634	9.63 × 10^−5^	up	negative
106	(+/−)11(12)-EET	C_20_H_32_O_3_	320.23546	8.216	319.22818	9.93 × 10^−5^	up	negative
107	1-[(3S)-3-(1,3-Benzoxazol-2-yl)-1-pyrrolidinyl]-3-methoxy-1-propanone	C_15_H_18_N_2_O_3_	274.13575	5.362	275.14301	0.000102	up	positive
108	Estrone	C_18_H_22_O_2_	270.16277	4.84	271.17004	0.000105	down	positive
109	L-(-)-Malicacid	C_4_H_6_O_5_	134.02165	1.651	133.01437	0.00011	down	negative
110	1,4-dihydroxyheptadec-16-en-2-ylacetate	C_19_H_36_O_4_	328.26148	9.25	351.25069	0.000113	down	positive
111	4-amino-6-chloro-3-cinnolinecarboxamide	C_9_H_7_ClN_4_O	111.01374	1.589	223.03451	0.000117	down	positive
112	Indoxylsulfuricacid	C_8_H_7_NO_4_S	213.00984	5.195	212.00256	0.000122	up	negative
113	WNH	C_21_H_25_N_7_O_5_	455.20205	4.721	456.20933	0.000128	down	positive
114	2-Arachidonoylglycerol	C_23_H_38_O_4_	378.27477	10.621	379.28204	0.000134	down	positive
115	12-Hydroxydodecanoicacid	C_12_H_24_O_3_	216.17279	6.982	215.16551	0.000136	down	negative
116	Oleanolicacid	C_30_H_48_O_3_	478.34248	9.008	479.34976	0.000142	up	positive
117	ProstaglandinF1β	C_20_H_36_O_5_	392.23359	11.278	391.22632	0.000156	up	negative
118	DLK	C_16_H_30_N_4_O_6_	374.21719	2.92	188.11596	0.000158	down	positive
119	N1-methyl-5-methoxy-2-({2-[(methylamino)carbonyl]phenyl}thio)benzamide	C_17_H_18_N_2_O_3_S	366.08343	5.018	365.07616	0.000178	down	negative
120	Stearamide	C_18_H_37_NO	283.28694	7.68	284.29419	0.000179	down	positive
121	NicotinuricAcid	C_8_H_8_N_2_O_3_	180.05369	1.69	179.04642	0.00018	up	negative
122	o-Veratraldehyde	C_9_H_10_O_3_	166.0636	5.049	184.09743	0.000183	up	positive
123	Epinephrine	C_9_H_13_NO_3_	183.09019	5.049	184.09747	0.000183	up	positive
124	Tetracycline	C_22_H_24_N_2_O_8_	444.15789	8.14	443.15061	0.000201	up	negative
125	CAR16:1	C_23_H_44_NO_4_	397.31839	7.664	398.32567	0.000204	down	positive
126	LPI14:0	C_23_H_45_O_12_P	544.26608	8.813	543.2588	0.000216	up	negative
127	2-Aminoadipicacid	C_6_H_11_NO_4_	161.06904	1.545	162.0763	0.000228	down	positive
128	Histamine	C_5_H_9_N_3_	111.07998	1.515	112.08726	0.000244	down	positive
129	2,4-dihydroxyheptadec-16-en-1-ylacetate	C_19_H_36_O_4_	350.24308	9.094	351.25036	0.000253	down	positive
130	S-Lactoyglutathione	C_13_H_21_N_3_O_8_S	379.10574	4.814	380.11301	0.000256	up	positive
131	Propionylcarnitine	C_10_H_19_NO_4_	217.13169	3.356	218.13897	0.000261	down	positive
132	Octadecanamine	C_18_H_39_N	269.30801	8.64	270.31528	0.000286	up	positive
133	ProstaglandinA3	C_20_H_28_O_4_	332.19935	6.942	331.19207	0.000287	down	negative
134	Gamma-Glu-Leu	C_11_H_20_N_2_O_5_	260.13613	7.471	261.14341	0.00033	down	positive
135	Dithranol	C_14_H_10_O_3_	226.06402	5.616	227.07091	0.000339	up	positive
136	Lithocholicacid	C_24_H_40_O_3_	422.30162	11.084	421.29434	0.000342	up	negative
137	N4-(4-chloro-2,5-dimethoxyphenyl)morpholine-4-carbothioamide	C_13_H_17_ClN_2_O_3_S	354.01766	1.364	377.00681	0.000344	down	positive
138	methyl3-(6-methylpyridin-2-yl)-2,2-diphenylpropanoate	C_22_H_21_NO_2_	331.15717	5.452	332.16445	0.000374	up	positive
139	LPI16:1	C_25_H_47_O_12_P	570.28161	9.137	569.27433	0.000389	up	negative
140	cis-Aconiticacid	C_6_H_6_O_6_	174.0166	1.963	173.00932	0.000393	down	negative
141	2-Methylbutyroylcarnitine	C_12_H_23_NO_4_	245.16328	5.709	244.15601	0.000404	down	negative
142	EMK	C_16_H_30_N_4_O_6_S	406.18623	5.072	407.19351	0.000408	down	positive
143	6-Deoxy-D-glucose	C_6_H_12_O_5_	164.06843	1.556	199.03783	0.000505	down	negative
144	2-hydroxy-3,6-diphenylcyclohexylacetate	C_20_H_22_O_3_	332.14082	5.747	333.14809	0.000512	up	positive
145	All-Trans-13,14-Dihydroretinol	C_20_H_32_O	288.24559	9.872	289.25277	0.000528	down	positive
146	5-Sulfosalicylicacid	C_7_H_6_O_6_S	217.98878	4.813	216.98151	0.000533	down	negative
147	N-Acetyl-1-aspartylglutamicacid	C_11_H_16_N_2_O_8_	304.09117	2.148	303.08399	0.000537	up	negative
148	Androsterone	C_19_H_30_O_2_	272.21401	9.952	255.2108	0.000595	up	positive
149	N-Acetylcysteine	C_5_H_9_NO_3_S	163.0306	3.487	162.02332	0.000632	up	negative
150	2-Aminobenzenesulfonicacid	C_6_H_7_NO_3_S	173.01534	1.708	191.0488	0.000672	up	positive
151	N8-Acetylspermidine	C_9_H_21_N_3_O	170.14228	1.359	188.17613	0.000734	down	positive
152	Uridinemonophosphate(UMP)	C_9_H_13_N_2_O_9_P	324.03615	1.734	323.02888	0.000761	down	negative
153	Tetrahydrocorticosterone	C_21_H_34_O_4_	350.24428	8.29	349.237	0.000795	down	negative
154	GLK	C_14_H_28_N_4_O_4_	316.21178	2.271	159.1132	0.000843	down	positive
155	MAG(18:3)	C_21_H_36_O_4_	352.26118	9.05	375.25045	0.000877	down	positive
156	Pantothenicacid	C_9_H_17_NO_5_	219.11078	5.05	218.10349	0.000979	up	negative
157	5-Phenyl-N-(4-(trifluoromethyl)phenyl)oxazol-2-amine	C_16_H_11_F_3_N_2_O	304.07988	5.086	303.0726	0.001002	down	negative
158	PB-22 N-(4-Hydroxypentyl)-3-carboxyindolemetabolite	C_14_H_17_NO_3_	229.11413	5.64	230.12141	0.001009	up	positive
159	Abametapir	C_12_H_12_N_2_	184.10031	5.399	183.09304	0.00106	up	negative
160	VitaminA	C_20_H_30_O	286.22989	9.341	287.23712	0.001146	down	positive
161	3-Hydroxy-3-methylglutaricacid	C_6_H_10_O_5_	162.05305	3.244	161.04577	0.001209	up	negative
162	4-Hydroxyisoleucine	C_6_H_13_NO_3_	147.08976	5.048	146.08249	0.001217	up	negative
163	3-Oxo-7alpha,12alpha-hydroxy-5beta-cholanoicacid	C_24_H_38_O_5_	406.27176	8.655	429.26102	0.00127	up	positive
164	Ozagrel	C_13_H_12_N_2_O_2_	228.09032	5.398	227.08311	0.001283	up	negative
165	13Z,16Z-DocosadienoicAcid	C_22_H_40_O_2_	336.30336	11.759	335.29608	0.001338	down	negative
166	Daidzein	C_15_H_10_O_4_	254.05843	5.322	253.05115	0.001368	up	negative
167	L-Asparticacid	C_4_H_7_NO_4_	133.03775	1.439	132.03047	0.001621	down	negative
168	UDP-N-acetylglucosamine	C_17_H_27_N_3_O_17_P_2_	607.08328	2.08	606.07601	0.00163	up	negative
169	CAR18:2	C_25_H_46_NO_4_	423.33385	7.927	424.34113	0.001801	down	positive
170	Docosanamide	C_22_H_45_NO	339.34967	8.113	340.35695	0.001868	up	positive
171	3-hydroxy-3-methylpentanedioicacid	C_6_H_10_O_5_	179.08096	4.901	180.08824	0.001953	down	positive
172	L-Malate	C_4_H_6_O_5_	134.0217	2.222	133.01442	0.00199	down	negative
173	D-Fructose6-phosphate	C_6_H_13_O_9_P	260.02923	5.552	259.02196	0.002423	up	negative
174	Lysops22:6	C_28_H_44_NO_9_P	569.27426	8.875	568.26699	0.002662	up	negative
175	CAR18:1	C_25_H_48_NO_4_	425.35002	8.295	426.35731	0.002684	down	positive
176	ProstaglandinE2	C_20_H_32_O_5_	352.2256	6.444	351.21833	0.002936	down	negative
177	DL-Malicacid	C_4_H_6_O_5_	134.02166	1.535	133.01437	0.002944	down	negative
178	15-OxoEDE	C_20_H_34_O_3_	322.25119	8.7	321.24391	0.003066	down	negative
179	Ergosterol	C_28_H_44_O	396.34013	9.972	397.3474	0.00308	up	positive
180	7-Methylguanosine	C_11_H_15_N_5_O_5_	297.10783	4.736	298.1151	0.003244	up	positive
181	5,8-dihydroxy-10-methyl-5,8,9,10-tetrahydro-2H-oxecin-2-one	C_10_H_14_O_4_	220.06773	5.938	463.12414	0.003308	up	positive
182	3-{[(4-fluorophenyl)sulfonyl]amino}-5-phenylthiophene-2-carboxamide	C_17_H_13_FN_2_O_3_S_2_	398.0202	5.67	399.02747	0.00339	down	positive
183	Uridine5′-monophosphate	C_9_H_13_N_2_O_9_P	324.03576	1.506	325.04304	0.003449	down	positive
184	Ferulicacid	C_10_H_10_O_4_	194.05835	5.749	193.05107	0.003609	up	negative
185	Palmitoylsphingomyelin	C_39_H_79_N_2_O_6_P	702.56812	11.165	703.57542	0.003775	down	positive
186	Tomatidine	C_27_H_45_NO_2_	415.34254	10.716	416.34982	0.003785	down	positive
187	LPG18:3	C_24_H_43_O_9_P	506.26537	8.712	505.25809	0.003816	up	negative
188	Fumaricacid	C_4_H_4_O_4_	116.01116	1.53	115.00388	0.003882	down	negative
189	5-OxoETE	C_20_H_30_O_3_	318.21584	8.573	319.22311	0.004151	up	positive
190	PC32:1	C_40_H_78_NO_8_P	753.52888	11.487	754.53615	0.004193	down	positive
191	LPH	C_17_H_27_N_5_O_4_	365.20767	4.864	183.61111	0.004262	down	positive
192	N2-tetrahydrofuran-2-ylmethyl-4-(4-fluorophenyl)-1,3-thiazol-2-amine	C_14_H_15_FN_2_OS	278.0942	4.887	279.10148	0.004346	up	positive
193	2,3-Dinor-8-epi-prostaglandinF2α	C_18_H_30_O_5_	326.20994	6.702	325.20266	0.004383	down	negative
194	Xanthosine	C_10_H_12_N_4_O_6_	284.07609	4.58	283.06881	0.004546	up	negative
195	Androsteroneglucuronide	C_25_H_38_O_8_	466.26151	11.762	465.25423	0.004653	down	negative
196	ProstaglandinB2	C_20_H_30_O_4_	334.21499	6.446	333.20775	0.00472	down	negative
197	Pyridoxamine	C_8_H_12_N_2_O_2_	168.09007	1.709	167.0828	0.004866	up	negative
198	LysoPC12:1	C_20_H_36_NO_7_P	433.22331	10.361	434.23059	0.005142	up	positive
199	D-Sphingosine	C_18_H_37_NO_2_	281.27149	7.738	282.27877	0.005404	down	positive
200	SPB19:1;2O	C_19_H_39_NO_2_	313.29764	7.958	296.29454	0.005472	down	positive
201	25-hydroxycholecalciferol	C_27_H_44_O_2_	400.33418	9.891	401.34147	0.005683	up	positive
202	Tretinoin	C_20_H_28_O_2_	300.20928	9.096	299.20201	0.005714	down	negative
203	SPB16:1;2O	C_16_H_33_NO_2_	271.25058	7.139	254.24738	0.00605	down	positive
204	(±)7(8)-DiHDPA	C_22_H_34_O_4_	362.24388	10.536	361.23661	0.006437	down	negative
205	LPE22:1	C_27_H_54_NO_7_P	535.36437	11.178	534.35709	0.006455	down	negative
206	Palmitoylethanolamide	C_18_H_37_NO_2_	299.28202	7.513	300.28935	0.006565	down	positive
207	L-Ascorbate	C_6_H_8_O_6_	176.03236	2.671	175.02506	0.006667	up	negative
208	AICAribonucleotide	C_9_H_15_N_4_O_8_P	300.10741	1.504	337.05604	0.006702	up	negative
209	dTMP	C_10_H_15_N_2_O_8_P	322.05653	2.247	323.06429	0.006993	down	positive
210	Nervonicacid	C_24_H_46_O_2_	366.3503	11.682	365.34302	0.007003	down	negative
211	XMP	C_10_H_13_N_4_O_9_P	364.04263	1.528	363.03535	0.007068	up	negative
212	Tauroursodeoxycholicacid	C_26_H_45_NO_6_S	499.29183	8.037	498.28455	0.007212	up	negative
213	Guanine	C_5_H_5_N_5_O	151.04966	1.669	152.05694	0.007274	down	positive
214	Creatinephosphate	C_4_H_10_N_3_O_5_P	211.03577	1.486	212.04289	0.007724	down	positive
215	13,14-Dihydro-15-ketoProstaglandinE2	C_20_H_32_O_5_	352.22891	9.338	351.22163	0.007811	down	negative
216	UDP-galactose	C_15_H_24_N_2_O_17_P_2_	566.05562	1.835	565.04834	0.008	down	negative
217	2-phenyl-2,4,6,7-tetrahydrothiino[4,3-c]pyrazol-3-ol	C_12_H_12_N_2_OS	232.06915	5.845	233.07651	0.008108	down	positive
218	4-Methyl-5-thiazoleethanol	C_6_H_9_NOS	143.04103	4.82	144.04831	0.008803	up	positive
219	LPC18:3	C_26_H_48_NO_7_P	563.32363	8.55	562.31635	0.008812	up	negative
220	Phosphoethanolamine	C_2_H_8_NO_4_P	141.01938	3.489	140.0121	0.008994	down	negative
221	Deoxyribose5-Phosphate	C_5_H_11_O_7_P	214.02458	1.465	259.02277	0.009015	down	negative
222	LPI18:1	C_27_H_51_O_12_P	598.31322	10.219	597.30594	0.009165	up	negative
223	LPK	C_17_H_32_N_4_O_4_	356.24351	4.797	179.12907	0.009173	down	positive
224	LPE22:5	C_27_H_46_NO_7_P	527.30214	9.02	526.29487	0.009643	down	negative
225	Palmitoylcarnitine	C_23_H_45_NO_4_	399.33451	8.128	400.3418	0.009796	down	positive
226	2-chloro-6-(1,4-thiazinan-4-yl)benzonitrile	C_11_H_11_ClN_2_S	260.0147	5.936	261.02197	0.009826	down	positive
227	dCDP	C_9_H_15_N_3_O_10_P_2_	387.02491	1.804	386.01764	0.010204	down	negative
228	S-Lactoylglutathione	C_13_H_21_N_3_O_8_S	379.10423	3.572	380.11151	0.010466	down	positive
229	LPI17:1	C_26_H_49_O_12_P	584.29753	9.838	583.29026	0.010797	up	negative
230	Cholesterol	C_27_H_46_O	386.3545	11.396	387.36161	0.011521	down	positive
231	Gly-Tyr	C_11_H_14_N_2_O_4_	238.09573	4.39	239.10301	0.012216	down	positive
232	Cholest-4-en-3-one	C_27_H_44_O	384.33957	11.308	385.34707	0.01228	down	positive
233	D-Glucose6-phosphate	C_6_H_13_O_9_P	260.03008	1.24	259.0228	0.012542	down	negative
234	morphine-d3	C_17_H_16_[2]H_3_NO_3_	271.12449	5.532	272.132	0.012548	up	positive
235	Dl-Glyceraldehyde3-phosphate	C_3_H_7_O_6_P	169.99808	1.412	192.98732	0.012554	down	positive
236	16(R)-HETE	C_20_H_32_O_3_	342.21464	9.446	343.22191	0.013386	up	positive
237	SM20:2;2O	C_25_H_49_N_2_O_6_P	526.31483	8.601	527.32211	0.013592	up	positive
238	3-{[(3S)-3-(1,3-Benzothiazol-2-yl)-1-pyrrolidinyl]methyl}benzonitrile	C_19_H_17_N_3_S	319.11752	5.325	320.12478	0.013719	down	positive
239	Mupirocin	C_26_H_44_O_9_	1044.56464	5.758	523.2896	0.013802	down	positive
240	S-(Methyl)Glutathione	C_11_H_19_N_3_O_6_S	321.09974	2.685	322.10702	0.013887	up	positive
241	L-Arabinitol	C_5_H_12_O_5_	152.06881	1.406	151.0615	0.014314	up	negative
242	gamma-Glutamylmethionine	C_10_H_18_N_2_O_5_S	278.09426	4.877	277.08699	0.014572	up	negative
243	PC16:0_16:1	C_40_H_78_NO_8_P	731.54681	11.524	732.55404	0.014578	down	positive
244	10-Nitrolinoleate	C_18_H_31_NO_4_	325.22584	6.354	324.21857	0.014608	down	negative
245	Uridine5′-diphosphoglucuronicacid	C_15_H_22_N_2_O_18_P_2_	580.03593	0.936	579.02866	0.014748	up	negative
246	13-HPODE	C_18_H_32_O_4_	312.22892	7.104	313.236	0.015141	up	positive
247	PE16:1_18:1	C_39_H_74_NO_8_P	715.51665	8.794	714.50937	0.016297	down	negative
248	4-(Diethylamino)salicylaldehyde	C_11_H_15_NO_2_	193.10845	5.563	194.11573	0.016762	down	positive
249	Docosapentaenoicacid	C_22_H_34_O_2_	330.25621	10.186	329.24893	0.017709	down	negative
250	Aminomalonicacid	C_3_H_5_NO_4_	119.02213	2.263	118.01486	0.018361	up	negative
251	WMH	C_22_H_28_N_6_O_4_S	472.19233	5.707	473.19961	0.018551	down	positive
252	3-(2,3-dihydro-1H-indol-1-yl)-2-[(2-furylmethyl)sulfonyl]acrylonitrile	C_16_H_14_N_2_O_3_S	314.07222	1.401	315.0795	0.018716	down	positive
253	2-Phenylacetamide	C_8_H_9_NO	135.06561	5.367	309.09378	0.019606	up	positive
254	Pizotifen	C_19_H_21_NS	295.1459	5.854	294.13862	0.02024	down	negative
255	4-acetyl-4-(ethoxycarbonyl)heptanedioicacid	C_12_H_18_O_7_	296.08372	5.655	297.09104	0.020248	down	positive
256	LPEO-18:1	C_23_H_48_NO_6_P	465.32294	10.063	464.31566	0.020855	up	negative
257	LPC14:1	C_22_H_44_NO_7_P	511.29184	7.885	510.28456	0.021171	up	negative
258	methyl6-{[4-(trifluoromethyl)anilino]carbonyl}nicotinate	C_15_H_11_F_3_N_2_O_3_	306.05724	4.518	307.06451	0.021742	up	positive
259	Cortodoxone	C_21_H_30_O_4_	346.21299	9.662	345.20571	0.023107	down	negative
260	β-Nicotinamidemononucleotide	C_11_H_15_N_2_O_8_P	334.05636	1.47	335.06363	0.023125	down	positive
261	N-Acetyl-L-phenylalanine	C_11_H_13_NO_3_	207.08986	5.653	206.08258	0.025062	up	negative
262	Rosuvastatin	C_22_H_28_FN_3_O_6_S	481.1694	4.899	480.16212	0.025277	down	negative
263	5-(6-hydroxy-6-methyloctyl)-2,5-dihydrofuran-2-one	C_13_H_22_O_3_	248.13969	6.019	249.14696	0.025292	down	positive
264	Pyrogallol	C_6_H_6_O_3_	126.03191	0.422	127.03918	0.025859	down	positive
265	2-[5-(2-hydroxypropyl)oxolan-2-yl]propanoicacid	C_10_H_18_O_4_	240.07218	2.532	241.07956	0.025902	down	positive
266	Pantetheine	C_11_H_22_N_2_O_4_S	278.13077	5.181	277.12306	0.026244	down	negative
267	GTP	C_10_H_16_N_5_O_14_P_3_	522.99019	1.585	521.98291	0.026991	down	negative
268	LPS16:1	C_22_H_42_NO_9_P	495.26066	8.855	494.25338	0.027007	up	negative
269	N-Acetyl-L-leucine	C_8_H_15_NO_3_	173.10543	2.098	174.11271	0.027241	down	positive
270	LPE20:5	C_25_H_42_NO_7_P	499.27084	8.511	498.26356	0.028403	down	negative
271	D-Mannose6-phosphate	C_6_H_13_O_9_P	278.04068	1.454	259.02284	0.028568	down	negative
272	(±)9-HpODE	C_18_H_32_O_4_	312.23009	10.662	311.22281	0.028898	down	negative
273	2,3-DinorprostaglandinE1	C_18_H_30_O_5_	308.19694	7.269	307.18967	0.029444	up	negative
274	CAR18:0	C_25_H_50_NO_4_	427.36593	8.758	428.3732	0.029497	down	positive
275	(2E,4E)-N-(2-methylpropyl)dodeca-2,4-dienamide	C_16_H_29_NO	251.22467	8.565	252.23213	0.030263	down	positive
276	(5-L-Glutamyl)-L-AminoAcid	C_8_H_14_N_2_O_5_	218.0905	1.757	217.08322	0.030331	down	negative
277	Lysopc16:1	C_24_H_48_NO_7_P	493.31792	9.154	492.31064	0.030434	up	negative
278	D-Homocysteine	C_4_H_9_NO_2_S	135.0356	1.539	136.04286	0.030734	up	positive
279	UMP	C_9_H_13_N_2_O_9_P	324.03662	1.633	347.02603	0.031106	down	positive
280	Corticosterone	C_21_H_30_O_4_	346.21876	10.906	347.22594	0.032826	down	positive
281	2-(3,4-dimethoxyphenyl)ethanamine	C_10_H_15_NO_2_	181.1107	5.7	226.1089	0.033212	up	negative
282	CAR20:1	C_27_H_52_NO_4_	453.38151	8.903	454.38877	0.033615	down	positive
283	2-hydroxy-6-[(8Z,11Z)-pentadeca-8,11,14-trien-1-yl]benzoicacid	C_22_H_30_O_3_	324.2042	10.077	325.21148	0.034219	down	positive
284	N-[1-(4-methoxy-2-oxo-2H-pyran-6-yl)-2-methylbutyl]acetamide	C_13_H_19_NO_4_	270.15711	8.886	271.16438	0.035764	down	positive
285	5-Methyl-2′-deoxycytidine	C_10_H_15_N_3_O_4_	241.10658	4.966	240.0993	0.036121	down	negative
286	2-Mercaptobenzothiazole	C_7_H_5_NS_2_	166.98653	6.003	165.97926	0.036641	down	negative
287	11(Z),14(Z),17(Z)-Eicosatrienoicacid	C_20_H_34_O_2_	306.2558	10.384	307.26317	0.0368	down	positive
288	N-Formylkynurenine	C_11_H_12_N_2_O_4_	236.08032	4.893	237.08759	0.037249	down	positive
289	ThromoboxaneB1	C_20_H_36_O_6_	408.22837	8.876	407.22109	0.038568	up	negative
290	diethyl2-{[2-(2-thienylcarbonyl)hydrazino]methylidene}malonate	C_13_H_16_N_2_O_5_S	350.03433	4.698	351.0416	0.040171	up	positive
291	LPG20:2	C_26_H_49_O_9_P	536.3124	11.26	535.30513	0.040667	down	negative
292	PEO-16:1_20:4	C_41_H_74_NO_7_P	723.52229	10.984	722.51501	0.042265	down	negative
293	α-Aspartylphenylalanine	C_13_H_16_N_2_O_5_	280.10644	5.298	281.11372	0.042952	down	positive
294	PEO-16:1_16:1	C_37_H_72_NO_7_P	673.50663	10.413	672.49935	0.04305	down	negative
295	2,4-Dinitrophenol	C_6_H_4_N_2_O_5_	184.01216	5.911	183.00488	0.043494	down	negative
296	Caffeine	C_8_H_10_N_4_O_2_	194.08059	5.35	195.08787	0.04419	down	positive
297	LPE20:1	C_25_H_50_NO_7_P	507.3332	10.382	506.32592	0.045014	down	negative
298	Adrenicacid	C_22_H_36_O_2_	332.27176	10.665	331.26449	0.045049	down	negative
299	1a,1b-DihomoprostaglandinF2α	C_22_H_38_O_5_	418.24932	11.807	417.24204	0.045094	up	negative
300	Adenosinetriphosphate(ATP)	C_10_H_16_N_5_O_13_P_3_	506.99653	1.555	505.98926	0.045309	down	negative
301	PC15:0_15:1	C_38_H_74_NO_8_P	703.51539	11.764	704.52275	0.047494	down	positive
302	6-(2-furyl)-2-hydroxy-4-(2-thienyl)nicotinonitrile	C_14_H_8_N_2_O_2_S	268.02827	5.044	267.02097	0.047838	up	negative
303	Lysopa18:0	C_21_H_43_O_7_P	438.27514	11.05	437.26786	0.048005	down	negative

**Table 3:** The significantly different pathways (*p* < 0.05) enriched the differentially abundant metabolites.

Pathway	*p* Value	Differential Metabolites	Polarity Ion Mode
Steroid hormone bio-synthesis	0.002248	Tetrahydrocorticosterone; Cortodoxone; Hydrocortisone; Andros-terone glucuronide; Adrenosterone	Negative
Steroid hormone bio-synthesis	0.038973	Cholesterol; Androsterone; Estrone; Dehydroepiandrosterone (DHEA); Corticosterone	Positive
Linoleic acid metabo-lism	0.044694	PC 32:1; (+/−)12(13)-DiHOME; 13-HPODE	Positive

## Data Availability

Data are contained within this article.
